# Editorial: Recent Advances in Myelin Plasticity

**DOI:** 10.3389/fncel.2021.688884

**Published:** 2021-05-21

**Authors:** Domna Karagogeos, Pascale Durbec, Kleopas Kleopa

**Affiliations:** ^1^School of Medicine, University of Crete, Heraklion, Greece; ^2^Institute of Molecular Biology and Biotechnology (IMBB), Foundation for Research and Technology Hellas (FORTH), Crete, Greece; ^3^UMR7288 Institut de Biologie du Développement de Marseille (IBDM), Marseille, Provence-Alpes-Côte d'Azur, France; ^4^The Cyprus Institute of Neurology and Genetics, Nicosia, Cyprus; ^5^School of Molecular Medicine, Cyprus Institute of Neurology and Genetics, Nicosia, Cyprus

**Keywords:** oligodendrocytes, microglia, astrocytes, plasticity, myelin

The Research Topic on Myelin Plasticity in the Non Neuronal Cells Section of the Frontiers in Cellular Neuroscience aims to provide an overview and new perspectives on the myelin formation and repair mechanisms. New insights into the cellular process and advances in technological tools in this field could reveal novel therapeutic strategies that may lead to the development of new treatments for central (CNS) and peripheral (PNS) nervous system de-/dys-myelinating pathologies. This is timely work given the high impact of CNS and PNS demyelinating disorders and is expected to contribute to the prevention of severe disabilities that have a serious impact on a patient's everyday life as well as on the society. Myelin plasticity is also a key mechanism that is involved in repair processes, a major challenge in human disorders.

The majority of myelin is formed postnatally in the rodents and by adulthood in humans. Although myelin plasticity in response to neuronal activity is an old observation, its extent has been appreciated relatively recently. It is now accepted that myelin can be shaped by environmental stimuli and undergo significant structural changes throughout life. This fine-tuning mechanism enhances neuronal function by orchestrating adjustments in myelin structure and axo-glial interactions. The potential link between this adaptive myelination and neuropsychiatric conditions is an active area of research.

In the special Research Topic, the review by Ronzano et al. summarizes how neuronal activity shapes the myelination profile during life emphasizing key parameters of the myelination process such as oligodendrocyte progenitor (OPC) proliferation, maintenance and differentiation, axon selection and myelination pattern. The authors examine myelination in adulthood as an adaptive mechanism and lastly but importantly, discuss how myelination and repair are also modulated by other glial cells such as astrocytes and microglia.

The review presented by Bonetto et al. focuses on myelin plasticity during adult life and described new insights into the link between this plasticity, learning and behavior, as well as mechanistic aspects of myelin formation that may underlie myelin plasticity, highlighting OPC diversity in the CNS.

Murphy et al. analyzes experience-dependent changes in myelin content in the adult visual and somatosensory cortex. Using models and conditions that drive adult plasticity and myelin basic protein (MBP) expression modulation, the authors demonstrated that induced plasticity can directly control visual activity in visual cortex. Using a deprivation visual paradigm, they showed that MBP content increased in the non-deprived hemisphere, while it decreased in the deprived hemisphere suggesting that modulation of myelin expression in adult visual cortex may reflect the levels of visually driven activity.

The review by Traiffort et al. focuses on the important contribution of astrocytes and microglia in the myelination process in normal and pathological conditions. The authors discuss the mechanisms by which astrocytes and microglia influence developmental myelination and their interplay with oligodendrocytes. They then proceed in discussing both beneficial and detrimental roles of these two glial cell types in de- and remyelination.

Petratos et al. analyze one of the key mechanisms at play during neuronal injury, namely the Nogo receptor A (NgR1)-mediated signaling cascade during myelination. Nogo is recognized as one of the major myelin-associated inhibitors but its role in maintaining axo-myelin stability and receptor binding is not completely elucidated. The authors provide recent insights as to how neuronal NgR1 regulates myelin thickness under normal and pathological conditions with particular emphasis on the regulation of perinodal domains. They further discuss how NgR1 signaling can be targeted in animal models as a future potential therapeutic strategy.

Deboux et al. have investigated the role of Slit1, a secreted axon guidance molecule also involved in adult neurogenesis, during myelin formation and in pathological condition. Using Slit1 deficient mouse, the authors showed that while Slit1 does not affect the normal developmental process of oligodendrogenesis and myelination, it regulates adult progenitor mobilization during remyelination by controlling cell migration and renewal within lesions.

Finally, El Waly et al. describe an innovative method to characterize the cascade of cellular events involved in lysolecithin (LPC)-induced demyelination by combining intravital coherent antistoke Raman scattering microscopy with intravital two-photon fluorescence microscopy in multicolor transgenic reporter mice. Taking advantage of spinal glass window implantation, a longitudinal description of cell dynamics during focal and reversible demyelination in the same volume of interest over weeks could be obtained. The authors detected early oligodendrocyte injury followed by axon degeneration within 2 days after LPC incubation, amplified by the recruitment of peripheral proinflammatory cells at day 4. Recovery took weeks and involved a new wave of anti-inflammatory innate immune cells at day 14. Overall, the use of recurrent imaging provided new insights into the role of peripheral immune cells in regulating both the axonal and oligodendroglial fates during de- and remyelination.

In conclusion, this special issue hosted manuscripts that highlighted several aspects of myelin plasticity both as reviews of current literature but also as original research contributions. They all provide insights into how myelin can be shaped throughout life under physiological or pathological conditions. We hope this collection of articles would be a useful contribution to the myelin field.

## Author Contributions

All authors listed have made a substantial, direct and intellectual contribution to the work, and approved it for publication.

## Conflict of Interest

The authors declare that the research was conducted in the absence of any commercial or financial relationships that could be construed as a potential conflict of interest.

